# Landau–Kleffner Syndrome Can Herald the Diagnosis of GRIN2A Gene Mutation

**DOI:** 10.1155/crpe/8869587

**Published:** 2025-07-20

**Authors:** Ayman Khalil Ebrahim, Jaafar Jawad Makhlooq, Maryam Yusuf Busehail

**Affiliations:** ^1^Pediatric Neurologist, Salmaniya Medical Complex, Rd No 2904, Manama, Bahrain; ^2^Pediatric Resident, Salmaniya Medical Complex, Rd No 2904, Manama, Bahrain; ^3^Pediatric Genetics, Salmaniya Medical Complex, Rd No 2904, Manama, Bahrain

## Abstract

Landau–Kleffner syndrome is a rare age-related childhood epileptic syndrome of linguistic decline and neuropsychological abnormalities as main clinical symptoms. It is a functional language disorder of children, manifesting with auditory verbal agnosia and other predominantly linguistic deficits. Also, cognitive and neurophysiological–behavioural abnormalities might manifest. Clinical seizures have been reported in three-quarters of children. Recent advances in genomic studies have provided important insights into the understanding of neurodevelopmental disorders such as autistic spectrum disorder, intellectual disability, and epilepsy. N-methyl-D-aspartate receptors (NMDARs) are glutamate-gated channels that are essential for synaptic transmission and plasticity in the central nervous system. Impaired NMDAR signaling due to genetic mutation causes a constellation of neurodevelopmental disorders that manifest as intellectual disability, epilepsy, and schizophrenia. A mutation in the GRIN gene which encodes NMDAR subunits can disrupt NMDAR function. In this article, we describe a 5-year-old boy who presented with aphasia and autistic- like behavior; during evaluation, subtle myoclonic jerks were noticed. Electroencephalogram revealed a hypsarrhythmia-like pattern, and following treatment with antiepileptic medications, he showed remarkable improvement in speech with better seizure control. Comprehensive genomic testing identified a heterozygous pathogenic variant in the GRIN2A gene. It is fundamental to maintain an awareness of the possible etiology of different epilepsy syndromes. Further description of this condition is detailed in this article.

## 1. Introduction

Epilepsy-aphasia syndromes (EASs) are a continuum of disorders that includes Landau–Kleffner syndrome, epileptic encephalopathy with continuous spike and wave during slow wave sleep (CSWS), atypical childhood epilepsy with centrotemporal spikes, and autosomal dominant Rolandic epilepsy with speech apraxia [1].

Age at onset is mainly before the age of six. Boys are twice as likely to be affected as girls. One or two cases are seen every year in highly specialized centers. This means that the prevalence of Landau–Kleffner syndrome under medical care is roughly one in 300,000 [2].

The prevalence of GRIN2A-related speech disorders and epilepsy in the general population is unknown; however, estimates can be made for some of the classic disorders.

GRIN2A pathogenic variants account for 9%–20% of EAS [3]. Genetic alteration of GRIN2A results in phenotype pleiotropy, predisposing to a broad range of epilepsy syndromes, with an elusive and unpredictable evolution and response to treatment [4].

## 2. The Case Report

A five-year-old boy presented with a 1-year history of unbalance gait, trembling, and frequent tripping. The boy was born by lower-segment cesarean section due to cephalopelvic dissociation. Pregnancy and delivery were uneventful. He achieved adequate motor development appropriate for his age, but his speech was delayed. He can say poorly intelligible short sentences. Over the last 1 year, the parents sought medical advice for his difficulty in walking, frequent falls, and worsening of his speech to the degree of mutism. The boy underwent extensive blood investigations including a metabolic screen which were unrevealing. The boy was later referred to the neurology service for further assessment. During evaluation, the boy was confused, not responding to verbal communication. He was fixing and following, extraocular movements were full, and no facial asymmetry was noted. No gross dysmorphic features were seen. Head circumference was 52 cm (50^th^ centile), and his weight measured 25 kg (25^th^ centile). The boy was unable to stand or walk, with tremulousness of the extremities. Motor examination for tone, power, and deep tendon reflexes was normal. Head drop was noticed during evaluation, and by further inquiring about minor seizures, the parents described some myoclonic jerks but denied any history of major motor seizures. At this point, the possibility of nonconvulsive status epilepticus or myoclonic astatic epilepsy was raised. Initial electroencephalogram study showed abnormal background activity with high amplitude, chaotic slow waves intermixed with sharp waves at central and frontal head regions, reminiscent to hypsarrhythmia pattern, and thus confirming the diagnosis of epilepsy and nonconvulsive status epilepticus. The boy was commenced on diazepam injections, loading dose of sodium valproate, followed by maintenance. A remarkable response to antiepileptic medication was noticed within a few days. The boy was more alert and attentive. After 6 weeks of treatment, the boy regained his baseline functions. He started walking alone and saying many words. His elder brother was diagnosed earlier with epilepsy but had poor adherence to antiepileptic medications. His electroencephalogram also showed generalized paroxysmal activity; he joined regular school but with poor academic performance. Given the familial nature of their epilepsy, genetic testing revealed a heterozygous pathogenic variant in the GRIN2A gene, c.1553G > A p.(Arg518His).

## 3. Discussion

In one study, the majority of cases (92.1%) had speech problems when they were first examined [5]. It was discovered to cover a broad range, from aphasia to mild speech and language impairment [6]. Similar to this, our patient suffered speech delay from an early age and then experienced speech deterioration that led to the aphasia phase. Speech delay was detected in 18.6% of GRIN2A patients [5].

According to Strehlow et al., most of the patients experienced seizures. Of them, tonic–clonic, infantile spasm, and focal seizures accounted for about half of the cases. A tiny portion did not experience seizures.

Four GRIN2A gene instances have been described by Liu et al. There have been reports of absence, myoclonic, atonic, and generalized tonic–clonic seizures. In addition, all demonstrated a favorable response to antiepileptic medications.

Similar to this, our patient experienced myoclonic jerks, whereas his older brother experienced generalized tonic–clonic seizures that responded well to antiepileptic drugs.

Roughly 6% of EEG records in Strehlow et al. were considered normal records. While the others showed abnormal discharges, about half of them were focal and one-third had CSWS. Liu et al., observed that the EEG transformed into 3 Hz or 3–5 Hz generalized spike and polyspike-and-sharp waves. Bilateral centrotemporal spikes and waves were also observed.

Our patient presented with an EEG that displayed a highly abnormal background with persistent sharp waves of high intensity suggestive of hypsarrhythmia and nonconvulsive epileptic status.

In contrast to the other patient, his EEG revealed bilateral centrotemporal sharp waves and generalized spike-wave complexes at 2–3 Hz.

His elder brother had a particular intellectual disability that resulted in poor academic performance. The range of intelligence and development reported by Liu et al. and Strehlow et al. is from normal development to learning disability. About one-third showed normal intelligence, while the remaining had intellectually disability, ranging from mild in 20% to profound in 9% [5].

The GRIN2A mutation is not present in all cases of EAS. Out of 44 patients with EAS that Carvell et al. tested, only four had GRIN2A.

Certain genetic mutations have been discovered to show a correlation between a mild intellectual impairment and the p.(Arg518His) missense mutation.

The genetic mutation in both patients was due to a missense mutation c.1553G > A p.(Arg518His) causing an amino acid change from Arg to His at Position 518. Pierson et al. have described a missense mutation of GRIN2A (NM_000833.3:c.2434C > A), resulting in changing leucine to methionine [7].

Another study has published the genetic results of four patients. Missense mutations included the following: c.2278G > A (p.G760S), c.4153G > T (p.D1385Y), c.1364G > A (p.C455Y), and c.691T > C (p.C231R) [8].

Lesca et al. have a publication in one article on 10 families and seven sporadic cases of GRIN2A genetic mutation. One family had a similar missense mutation as our patients p.(Arg518His), while the others showed different mutations [9].

## 4. Conclusion

GRIN2A gene mutation has been observed in some cases of EAS, and it might be associated with intellectual disabilities and learning difficulties. Furthermore, seizure semiology may differ from one patient to another.

## Data Availability

The data that support the findings of this study are available from the corresponding author upon reasonable request in both the system and the manuscript file.

## References

[1] Turner S. J., Morgan A. T., Perez E. R., Scheffer I. E. (2015). New Genes for Focal Epilepsies With Speech and Language Disorders. *Current Neurology and Neuroscience Reports*.

[2] Kaga M., Inagaki M., Ohta R. (2014). Epidemiological Study of Landau-Kleffner Syndrome (Lks) in Japan. *Brain and Development*.

[3] Carvill G. L., Regan B. M., Yendle S. C. (2013). GRIN2A Mutations Cause Epilepsy-Aphasia Spectrum Disorders. *Nature Genetics*.

[4] Gheța I., Teleanu R. I., Roza E., Carapancea E., Vladacenco O., Teleanu D. M. (2021). GRIN2A Variant in a 3-Year-Old-An Expanding Spectrum?. *Neurology International*.

[5] Strehlow V., Heyne H. O., Vlaskamp D. R. M. (2019). *GRIN2A*-Related Disorders: Genotype and Functional Consequence Predict Phenotype. *Brain*.

[6] Liu X. R., Xu X. X., Lin S. M. (2021). *GRIN2A* Variants Associated With Idiopathic Generalized Epilepsies. *Frontiers in Molecular Neuroscience*.

[7] Pierson T. M., Yuan H., Marsh E. D. (2014). *GRIN2A* Mutation and Early-Onset Epileptic Encephalopathy: Personalized Therapy With Memantine. *Annals of Clinical and Translational Neurology*.

[8] Qian P., Yang X., Xu X., Liu X., Zhang Y., Yang Z. (2018). *Study of GRIN2A Mutation in Epilepsy-Aphasia Spectrum Disorders*.

[9] Lesca G., Rudolf G., Bruneau N. (2013). GRIN2A Mutations in Acquired Epileptic Aphasia and Related Childhood Focal Epilepsies and Encephalopathies With Speech and Language Dysfunction. *Nature Genetics*.

